# Thrombin@Fe_3_O_4_ nanoparticles for use as a hemostatic agent in internal bleeding

**DOI:** 10.1038/s41598-017-18665-4

**Published:** 2018-01-10

**Authors:** Emiliya M. Shabanova, Andrey S. Drozdov, Anna F. Fakhardo, Ivan P. Dudanov, Marina S. Kovalchuk, Vladimir V. Vinogradov

**Affiliations:** 1 ITMO University, Laboratory of Solution Chemistry of Advanced Materials and Technologies, Lomonosova St. 9, 191002 St. Petersburg, Russian Federation; 2 Mariinsky Hospital, Regional Cardiovascular Center, Liteyny Ave. 56, 191054 St. Petersburg, Russian Federation

**Keywords:** Magnetic materials, Drug delivery, Organic-inorganic nanostructures

## Abstract

Bleeding remains one of the main causes of premature mortality at present, with internal bleeding being the most dangerous case. In this paper, magnetic hemostatic nanoparticles are shown for the first time to assist in minimally invasive treatment of internal bleeding, implying the introduction directly into the circulatory system followed by localization in the bleeding zone due to the application of an external magnetic field. Nanoparticles were produced by entrapping human thrombin (THR) into a sol-gel derived magnetite matrix followed by grinding to sizes below 200 nm and subsequent colloidization. Prepared colloids show protrombotic activity and cause plasma coagulation in *in vitro* experiments. We also show here using a model blood vessel that the THR@ferria composite does not cause systematic thrombosis due to low activity, but being concentrated by an external magnetic field with simultaneous fibrinogen injection accelerates local hemostasis and stops the bleeding. For instance, a model vessel system with circulating blood at the puncture of the vessel wall and the application of a permanent magnetic field yielded a hemostasis time by a factor of 6.5 shorter than that observed for the control sample. Biocompatibility of composites was tested on HELF and HeLa cells and revealed no toxic effects.

## Introduction

Severe injuries are usually accompanied by hemorrhage, so early intervention to stop bleeding plays a decisive role in saving human lives. An ideal hemostatic agent must have a number of key parameters: high hemostasis efficiency, safety, availability class. These are mainly concerned with content, as opposed to appearance^1^. Despite the existence of a wide range of hemostatic drugs used in clinical practice, such as aprotinin, desmopressin, tranexamic acid, aminocaproic acid, their main drawback is the need for their local application. Low selectivity, short half-lives in the body and side effects prevent using them to stop internal bleeding, which stimulates the development of nanocomposite hemostatic agents. The greatest attention is paid to nanocomposites based on natural polymers (e.g., keratin, silica, chitosan) with immobilized proteins from the hemostasis system, as well as synthetic platelets^2–9^. Such systems have a prolonged time of action and an extended therapeutic window, but in general are lack selectivity. In order to perform targeted delivery of hemostatic agents further modification of their surface by targeting agents, such as RNAi^10^, phospholipids^11^, antibodies^12^ and others^13–15^. This strategy significantly increases hemostatic efficiency but also complicates the synthetic procedures. In order to simplify the system another targeting strategies could be applied, relying on physical stimuli. Magnetic targeting of nanoformulations by magnetically controlled composite materials based on magnetite is one of the most promising and fast developing area on nanomedicine. Magnetite due to its biocompatibility and low toxicity has already been approved by FDA as an MRI contrast agent (Feridex, Combidex) and for treatment of iron deficiency (anemia) in people with chronic kidney failure (Ferumoxytol, Feraheme). Magnetite-based hemostatic and wound-healing systems are being developed by modification with appropriate bioactive compounds. For instance, in previous studies a composite based on *γ*-Fe_2_O_3_ nanoparticles decorated with a BSA coating and immobilized thrombin was described and used in *in vivo* study as a wound healing material^16^. The physical conjugation of thrombin to the *γ*-Fe_2_O_3_ nanoparticles preserved the thrombin clotting activity and improved it by stabilizing the enzyme against its major inhibitor antithrombin III.Another example of ferria-based healing materials was shown in our previous work, where a number of different functional drugs were entrapped in magnetite films providing slow release and better healing efficiency with decreasing the scar size^17^. Magnetic proteolytic composites based on sol-gel magnetite and collagenase enzyme were shown to be potential agents for minimally invasive treatment of adhesive disease and Dupuytren’s contracture^18^. Nevertheless, we emphasize that to date there are no drugs allowing one to stop internal bleeding without the use of surgical intervention. The development of new drugs with minimally invasive action (injection into the required area) will significantly increase the possibility of therapy of internal bleeding and affect the disappointing mortality statistics.

In this paper, we propose to consider the possibility of producing injectable magnetically controlled hemostatic drugs, whose purpose is a multi-fold increase in activity due to the drug localization at the selected point, i.e., in the puncture zone. Without the application of an external magnetic field, nanoparticles (with a size of less than 200 nm) freely propagate through the body and are metabolized. Thrombin was selected as an active substance, since it is important in the process of hemostasis (for converting fibrinogen into fibrin)^19^. In addition, thrombin is an activator of other hemostatic factors (factor VIII, factor V, factor XI, and factor XIII and protein C). In clinical practice, thrombin has been used to treat local hemostasis for many years^19,20^. In combination with fibrinogen, it enables skipping most stages of the coagulation mechanism, leading the process to the final stage of the coagulation cascade, so that two drugs quickly form a fibrin clot in the bleeding zone. As regards a method for creating a styptic magnetic colloid, we propose the use of entrapment methodology developed by us previously^21^ and successfully tested using the example of injectable thrombolytic drugs^22^.

We show here using coagulation time analysis in cuvettes and a model blood vessel that thrombin entrapped in magnetite not only does not cause systemic thrombosis in the blood due to low activity, but also is very capable of accelerating hemostasis when an external focused magnetic field with parallel fibrinogen injection is applied. E.g., for a model vessel system with circulating blood the time of hemostasis at the puncture of the vessel wall with the application of a permanent magnetic field was shorter by a factor of 6.5 as compared with the control sample. The obtained results evidence that the use of hemostatic drugs is possible not only when applied to the exterior of the vessel, but also from the inside, performing the function of blood cells that can also participate in the cascade of blood-hemostasis reactions, while providing an external control. The overall strategy and the study of the new material are carried out in conjunction with the previously proposed approach to the development of new materials for minimally invasive surgery^18,22,23^.

## Results and Discussion

### Synthesis and characterization of THR@ferria

**Figure 1:**
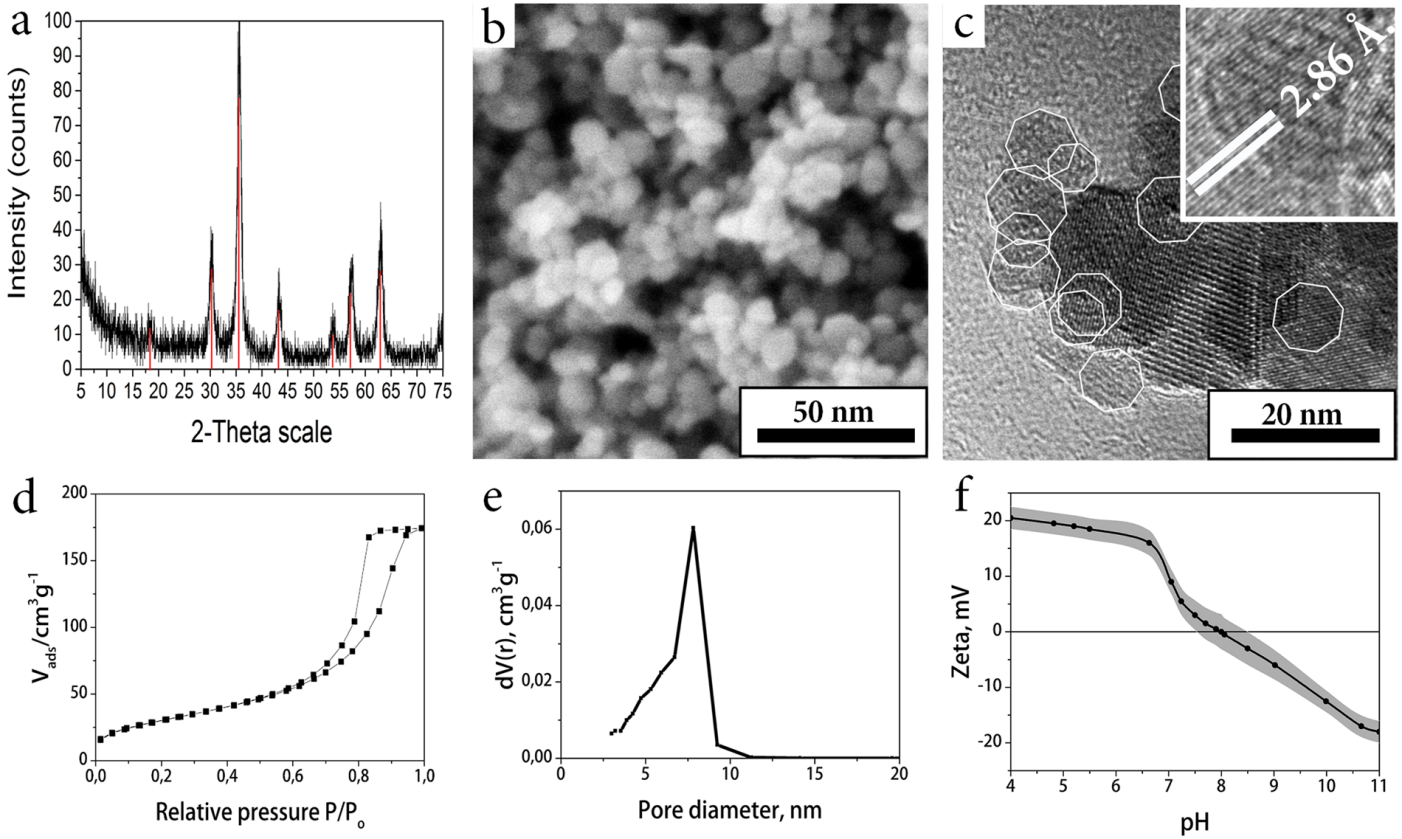
XRD diffraction pattern of the material corresponds to magnetite oxide phase, peaks referred to JCPDS file No. 19–0629: (red lines) (**a**); SEM image shows porous microstructure of the material (**b**); according to TEM, a magnetite matrix consists of truncated tetrahedron nanoparticles (highlighted by white contour) with narrow size distribution (**c**); low-temperature N_2_ physisorption curve shows mesoporous structure of the material (**d**); pore size distribution of the magnetite xerogel matrix by the BJH method (**e**); zeta potential of the magnetite matrix at different pH values. At pH = 7.4 the matrix is positively charged (**f**).

Hemostatic nanocomposites were produced by entrapment of widely used in clinical practice thrombin withing porous magnetite matrix in a course of room-temperature sol-gel transition. In order to do so a highly stable magnetite hydrosol was used, described earlier^24,25^. Careful optimization of the synthetic procedures allowed to produce hydrocolloids with high (up to 10% wt.) mass fractions of ferria nanoparticles, represented by magnetite phase (proved by FTIR, XRD and XPS analysis, for details see refs^24,25^. and ESI Figs S1 and S2). The key feature of the hydrosols is the excellent colloidal stability at neutral pH level and absence of any extraneous molecules on the surface of the nanoparticles. Due to synthetic conditions, the surface of the used magnetite nanoparticles contains a large number of hydroxyl groups, so that their isoelectric point is shifted to pH 8.2 and zeta potential at neutral pH values is sufficient to form a stable colloidal system (ESI Figs S3 and S4) Due to the lack of strong acids and bases in the hydrosol composition, it is compatible with enzyme molecules and does not cause their inactivation upon mixing^21^. Human thrombin was selected as a bioactive agent for producing a composite because of its structure and mechanism of action^26^. Thrombin is a globular protein from the family of serine proteases and is the main enzyme that triggers the thrombogenesis process in the body. During the formation of thrombus in the first stage, thrombin promotes partial lysis of the fibrinogen molecule, and two peptides A (molecular weight ~2000 Da) and two peptides B (molecular weight ~2500 Da) are cleaved from it to form a fibrin monomer constructed from two identical subunits connected by disulfide bonds^27^. In the second stage, the fibrin monomer spontaneously converts into a clot called a fibrin aggregate, or unstabilized fibrin. Aggregation of fibrin monomer (self-assembly of fibrin fibers) involves the transition of the molecule from the globule state to the fibril one. Hydrogen and electrostatic bonds and hydrophobic interaction forces take part in the formation of the fibrin aggregate. The formation of the fibrin aggregate is accelerated by substances carrying a positive charge (usually calcium ions, protamine sulfate), and is inhibited by negatively charged compounds (heparin)^28^. In the third stage, the fibrin aggregate undergoes changes due to the enzymatic action of the fibrin-stabilizing factor XIIIa (or fibrinoligase). Under the influence of this factor, strong covalent bonds between *γ*- and also between *α*-polypeptide chains of fibrin-aggregate molecules are formed, as a result of which it is stabilized into fibrin-polymer insoluble in media with high ionic strengths. Immobilization of thrombin was performed in a manner it was described earlier^21^ (for details see experimental part). For this purpose thrombin was entrapped within magnetite sol-gel matrix in a course of irreversible room-temperature sol-gel process. Condensation of magnetite hydrosol in a presence of thrombin solution results in formation of a mesoporous xerogel matrix in which the enzyme is entrapped (Fig. 1a–c). The matrix itself consists of truncated regular-shaped octahedra with an average diameter of 10 nm and has a developed porous nanoarchitecture (Fig. 1b,c), a specific surface area of 120 m^2^/g, and an average pore size of 8 nm (Fig. 1d,e). Due to the complementarity between the pore size and the thrombin molecule, the immobilized enzyme is firmly retained in the matrix pores. However, mass transfer allows the enzyme to interact with high-molecular substrate molecules according to our previous observations^18,22^.

The process of the composite formation involved two stages: interaction of thrombin with magnetite nanoparticles in colloid solution and the formation of rigid inorganic matrix. When the condensation process is carried out at neutral pH, the first stage involves the electrostatic interaction of magnetite nanoparticles having a positive charge of 30 mV (hydrosol isoelectric point is pH 8, Fig. 1f) with thrombin molecules having a negative charge of −6 mV (isoelectric point 6.3)^27^. Subsequent solvent removal led to formation of interparticle bonds between magnetite nanoparticles and formation of inorganic matrix doped by enzyme molecules immobilized in micropores of the material. The enzyme is immobilized rather physically than chemically, since no covalent bonds are formed between enzyme and inorganic matrix^21,29,30^. Entrapped enzyme is homogeneously distributed in the composite and can be located both inside the matrix and in its near-surface layer. An analysis of the electrostatic potential surface shows that upon entrapping thrombin will apparently affect a positively charged heparin binding site, located near the catalytic center, in a way to orient it on the maximum distance from the positively charged pore wall (ESI Fig. S5). In this orientation, the catalytic site will be oriented straight to the pore, through which the mass transfer is carried out, thus proving to be accessible to interaction with the substrate^21^.

**Figure 2:**
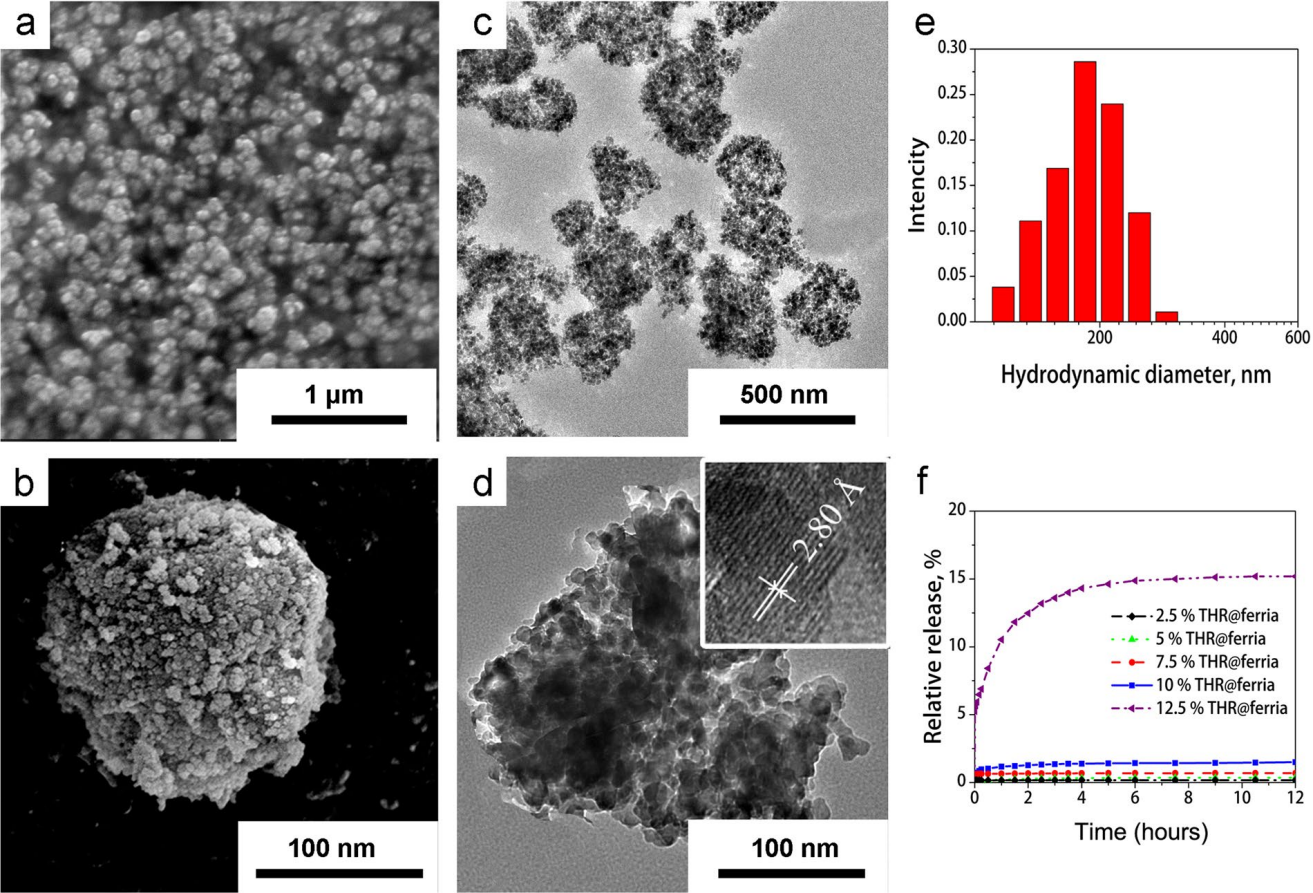
SEM (**a**,**b**) and TEM (**c**,**d**) images of THR@ferria NPs. Interplane spacing is presented in the insert. Hydrodynamic diameter of the composite NPs (**e**) and profile of thrombin release from them (**f**) For materials with thrombin mass fractions of less than 10% minor release is observed.

After condensation, the composite matrix was mechanically ground and transferred to a colloidal state by dispersion in saline solution and filtered through a syringe filter to eliminate particles with sizes larger than 200 nm, which is the limit allowed for intravenous administration (Fig. 2a–e) (see the synthesis diagram in experimental part). Evaluation of the release profile using the Bradford technique showed that for materials containing up to 10% thrombin, complete entrapment of thrombin into the xerogel matrix occurs and its release from the composite is not observed (Fig. 2f).

### THR@ferria cytotoxicity

**Figure 3:**
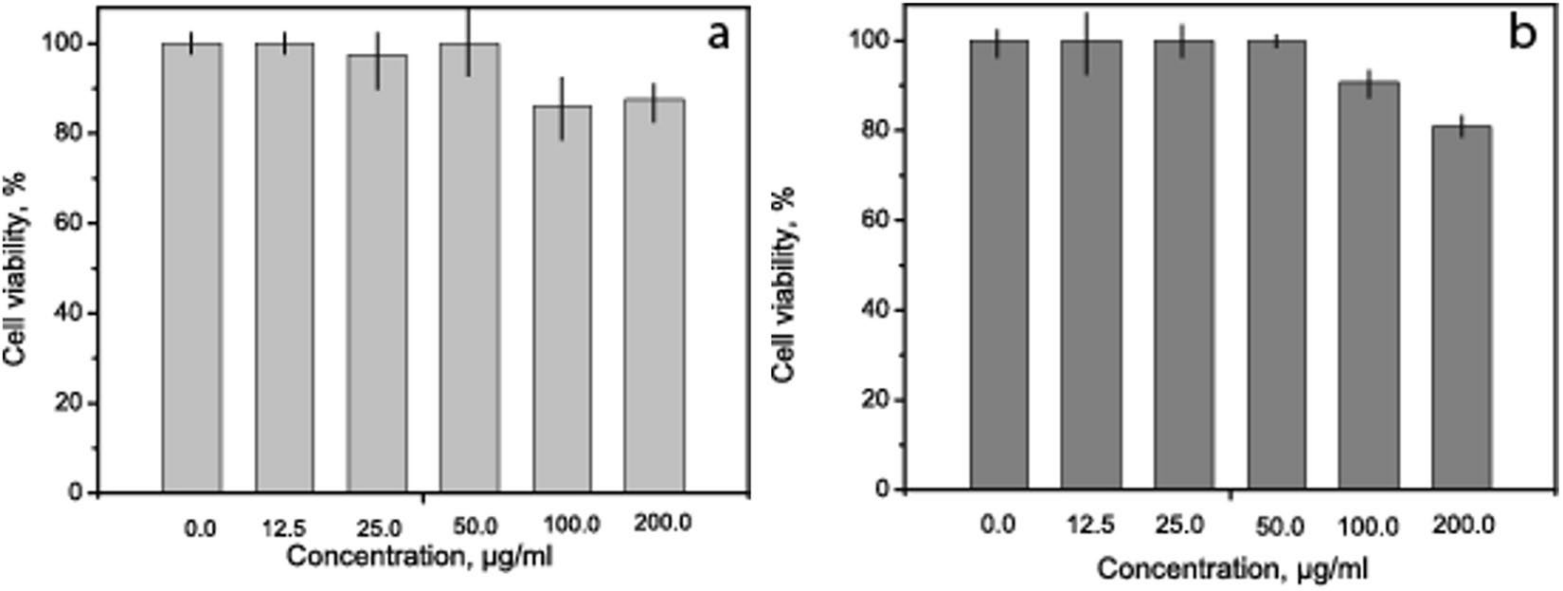
Cytotoxicity on (**a**) HeLa and (**b**) HELF cells after 72 h with THR@ferria NPs. Average values of 3 measurements with standard deviations are shown.

Before we proceed to the analysis of hemostatic activity, it is important to reveal that the synthesized nanoparticles possess low cytotoxicity. The cytotoxicity of magnetite and its compounds has been studied previously. It was shown that magnetite nanoparticles coated with triblock copolymers affect cell viability even in doses lower than 1 mg/mL because of toxic influence of polymers^31^. Other studies have reported that iron (II,III) oxide nanoparticles induce concentration-dependent cytotoxicity, appearing at doses higher than 2500 *μ*g/mL. Toxic effects of superparamagnetic iron oxide nanoparticles were rather low at concentrations up to 20 mM^32^. In another study, the cell viability decreased to 55% after the exposure to 200 *μ*g/mL superparamagnetic nanoparticles^33^. To evaluate biocompatibility of our NPs, we conducted cytotoxicity studies performed by the MTT assay (Fig. 3). Choice of maximum concentration was conditioned by the concentration of iron oxide nanoparticles in the stable sol (we used a 200-fold dilution of the initial sol in culture medium). THR@ferria (12.5–200 *μ*g/mL) was shown to have no cytotoxic effect on the Hela and HELF cells after 72 h exposure. Cell viability was 80.8% (for HELF cells) and 87.6% (for Hela cells), indicating that the composite NPs did not cause significant cell death and has low cyctotoxicity, comparable to results reported earlier.

### Coagulation activity of THR@ferria

The main goal of this work is the production of a composite capable of accelerating the hemostatic process by localizing directly at the site of internal bleeding. The blood coagulation system consists of very complex cascade reactions. The most important element of this system is thrombin, since it is responsible for the activity of a number of hemostatic factors (factor VIII, factor V, factor XI, and factor XIII and protein C). In addition, in combination with fibrinogen, thrombin enables skipping most of the steps of the coagulation mechanism, leading the process to the final stage of the coagulation cascade. Thus, we needed to check whether thrombin could retain its activity in an entrapped form. To this end, the activity of THR@ferria composite nanoparticles was tested on human plasma.

**Figure 4:**
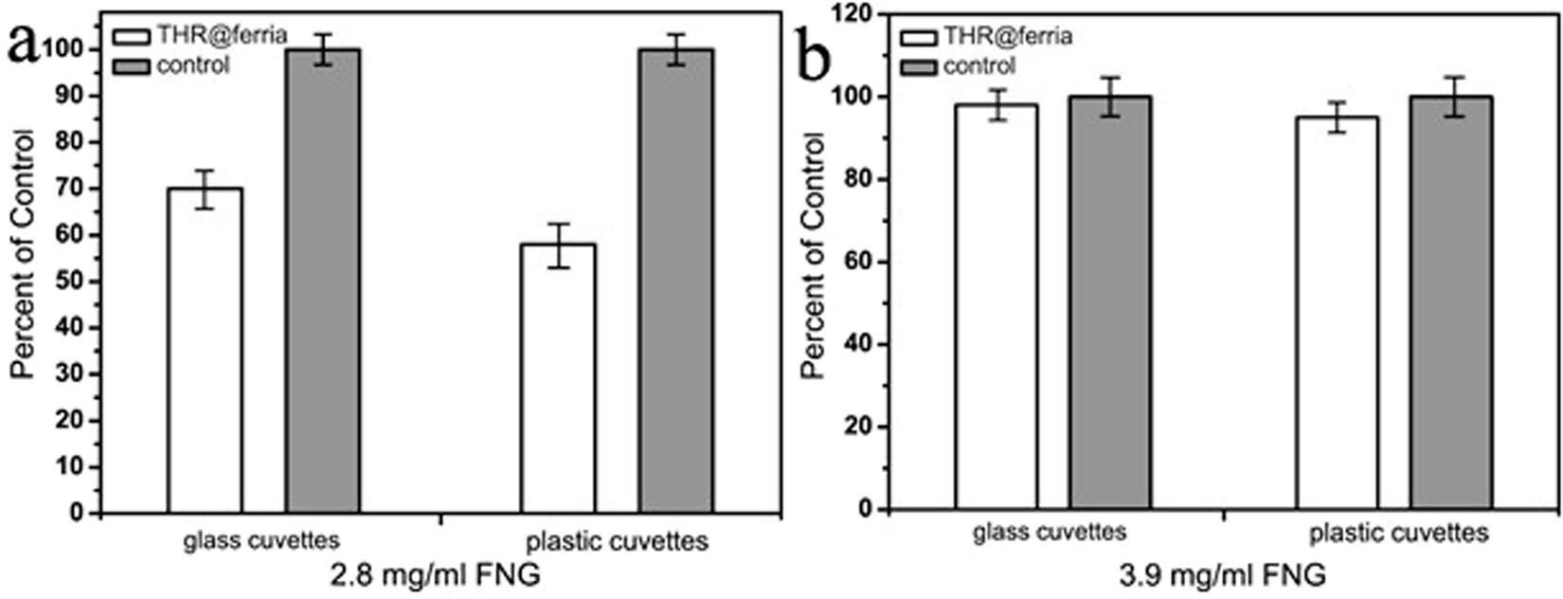
Coagulation time reported as percent of control both in glass and plastic cuvettes at fibrinogen concentrations of 2.8 mg/mL (**a**) and 3.9 mg/mL (**b**). The increased concentration of fibrinogen (**b**) is shown to yield comparable coagulation times for free and entrapped thrombin.

Coagulation time was evaluated in glass and plastic cuvettes to elucidate any direct effect on coagulation *in vitro* as glass is known to activate the contact activation pathway^34^. The effect of fibrinogen concentration in plasma was also assessed. To perform the experiments, a thrombus-forming agent was added to the standard human plasma and the thrombus formation time was determined by measuring the optical density (for details see experimental part). Results are summarized in Fig. 4. The experimental results suggest that when a sample of plasma is mixed with pure thrombin solution, coagulation occurs significantly (about 2 times) faster than in the case of THR@ferria (Fig. 4). The lower activity of the composite material is explained by the heterogeneity of the system, which leads to diffusion limitations of the process and a decrease in activity at similar thrombin concentrations in the system. However, when an additional volume of fibrinogen is introduced, comparable activity of free and entrapped thrombin is observed. As shown previously^35^, increasing the concentration of fibrinogen does not promote the initiation of thrombogenesis, but can significantly accelerate the coagulation time. Fibrinogen supplementation is currently considered as a therapeutic option for hemostatic management of trauma-related bleeding^36^.

The 2013 update of the European Trauma Guidelines recommends fibrinogen concentrate among the initial procoagulant therapies for patients with massive hemorrhage. One can assume that the localization of THR@ferria nanoparticles in the zone of internal hemorrhage due to a focused magnetic field with appropriate fibrinogen supplementation will significantly reduce the coagulation time in the required place without initiating systemic thrombus formation. Thus, preliminary experiments have shown that thrombin, while remaining in the matrix, retains the ability to hydrolyze four arginyl-glycyl peptide bonds in the fibrinogen molecule, which results in the formation of a fibrin monomer, the basis of the blood clot. Controlling activity of the entrapped enzyme can be achieved by regulating fibrinogen in the blood and, as will be shown in the next section, by focusing the drug at a local bleeding point.

### Analysis of hemostatic activity of THR@ferria

**Figure 5:**
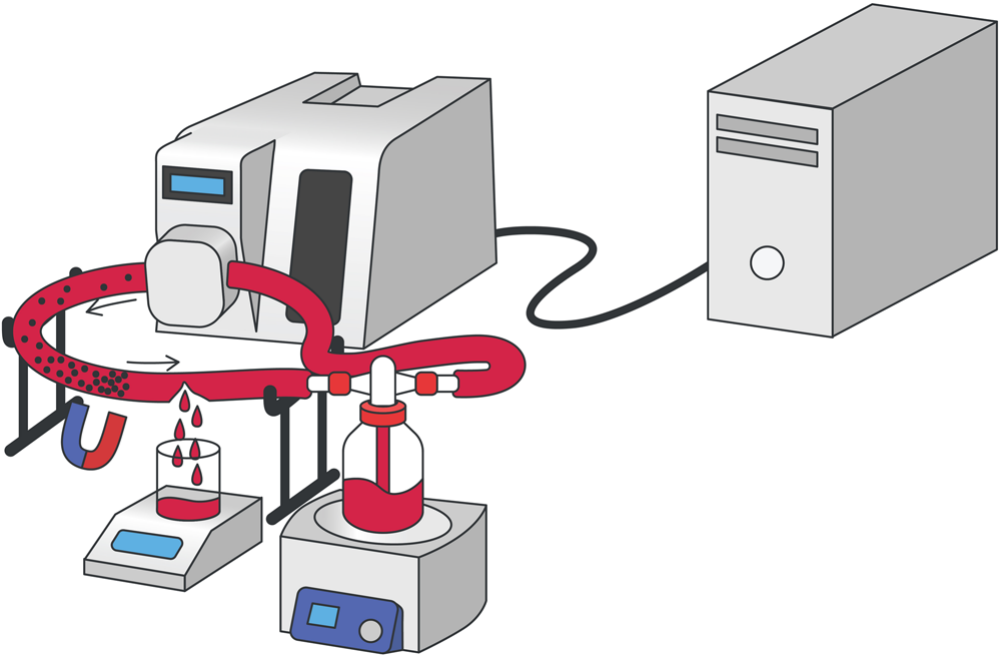
Installation scheme for assessing hemostatic effect using the synthesized THR@ferria NPs. A model stand with an artificial blood vessel is shown, inside which a colloid is introduced, followed by localization of nanoparticles at the site of bleeding due to the application of an external magnetic field.

The next stage implied testing the ability of synthesized nanoparticles to locally form a thrombus assisted by an external magnetic field in the model of an artificial vessel with circulating human blood without significantly altering the overall blood hemostasis. The circulation was provided with a peristaltic pump (see installation diagram in Fig. 5) and a transparent polyvinyl chloride tube with an internal diameter of 6 mm was chosen as a model vessel (see materials and methods section for experimental details). To determine the effectiveness of the hemostatic effect, the circulating blood stream was treated with 1 mL of a colloid consisting of THR@ferria nanoparticles with an activity of 10 NIH/mL, which were easily manageable when an external permanent magnetic field was applied.

**Figure 6:**
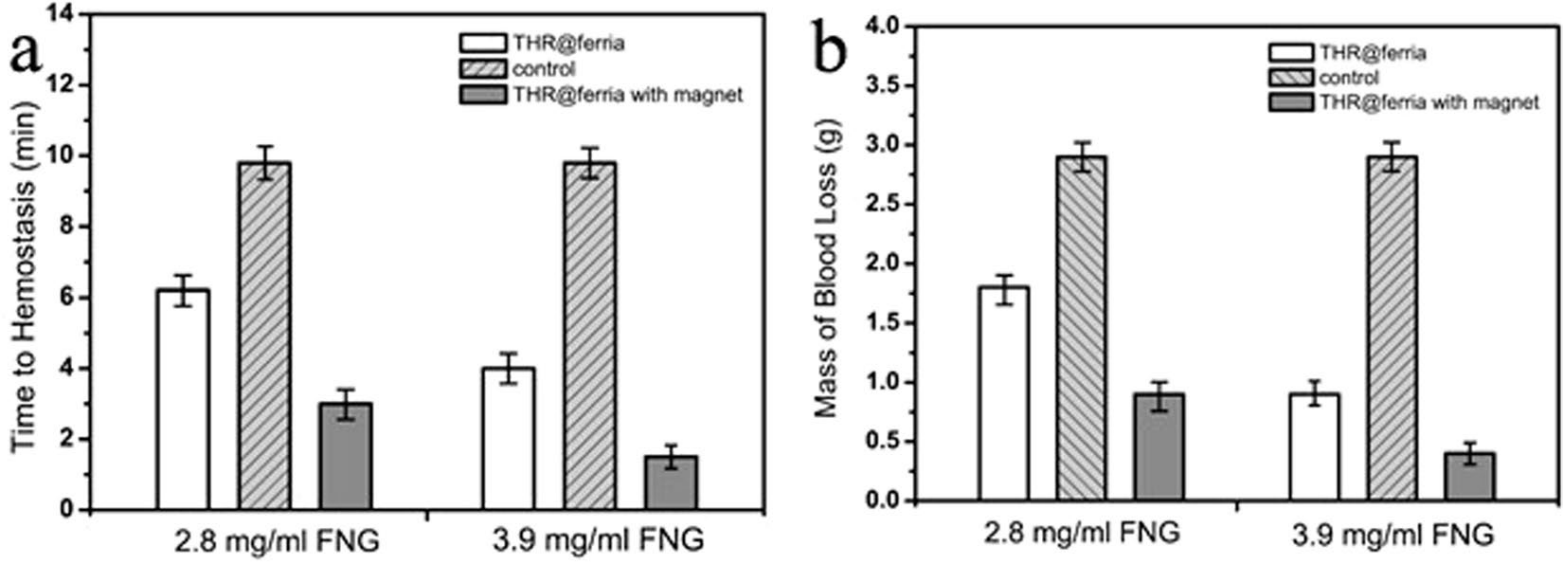
Time (**a**) and mass of blood (**b**) flowed out of the model blood vessel during an experiment on analyzing hemostatic activity. The measurements were carried out with different amounts of fibrinogen. The administration of hemostatic nanoparticles is shown to significantly reduce the time and mass of blood prior to hemostasis compared to the control, especially when an external magnetic field is applied and the nanoparticles are concentrated accordingly.

When a hole was made in the model vessel, the time and mass of blood loss were measured until the time of thrombus formation (end of bleeding) (Fig. 6). The obtained data indicate that the time to reach hemostasis for a system with magnetically induced nanoparticles appeared to shorter than that for the control sample by factors of 3.3 (for 2.8 mg/mL Fbr conditions) and 6.5 (for 3.9 mg/mL Fbr conditions). Even more outstanding data were obtained when measuring the mass of blood loss: 3.2 and 15.5 times smaller than those for the control sample with appropriate contents of fibrinogen in the blood. Based on the results of the experiment, it can be seen that the THR@ferria composite itself accelerates the process of hemostasis, but does not cause systemic coagulation of the blood. At the same time, its localization due to apposition of an external magnetic field leads to a tremendous increase in hemostatic activity in the required area, which opens up new horizons for minimally invasive hemostatic therapy.

**Figure 7:**
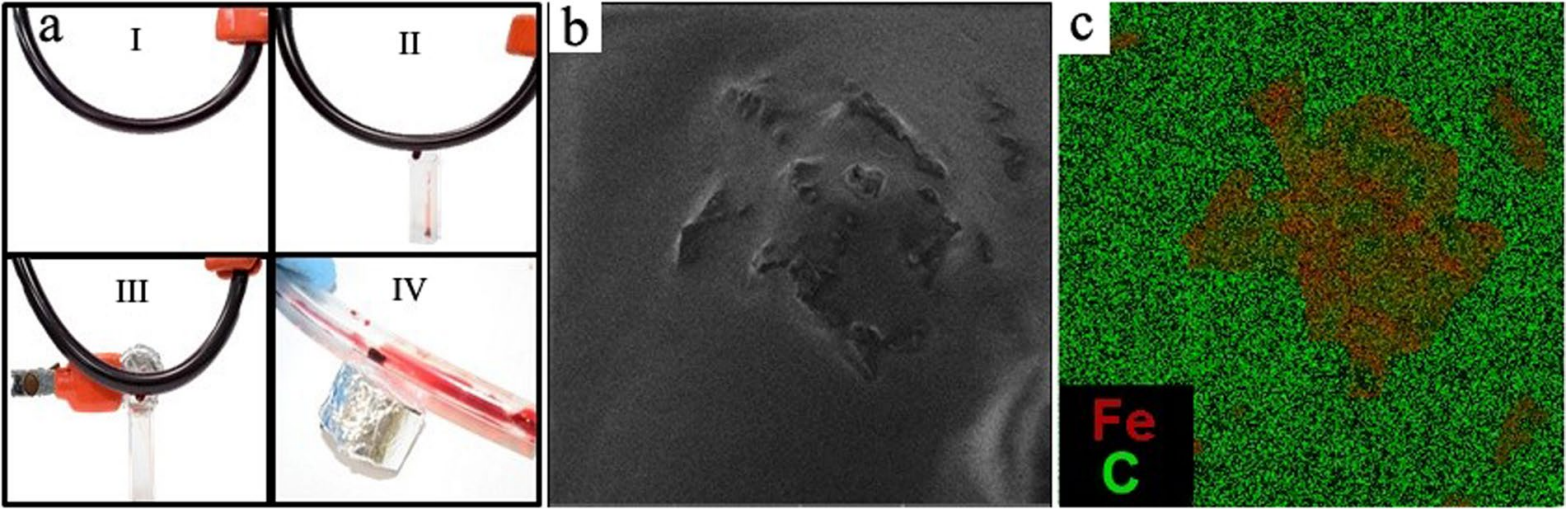
Analysis of hemostatic activity of THR@ferria: (**a**) General view of the model vessel with circulating blood and nanoparticles (I), bleeding after puncture (II), Magnet apposition to the bleeding zone (III) and appearance of the formed thrombus on the vessel wall after hemostasis. (**b**) SEM analysis of the thrombus surface with appropriate image mapping of the Fe content (**c**). The composite nanoparticles are shown to concentrate in the thrombus.

The appearance of the vessel in the experiment is shown in Fig. 7. It is clearly seen that after the hemostasis caused by concentrating nanoparticles at the site of bleeding, a parietal thrombus forms (Fig. 7a). An analysis of this thrombus using scanning electron microscopy has shown (Fig. 7b) that it contains a large amount of THR@ferria nanoparticles, which is confirmed by the result of monitoring the external surface of the thrombus, as well as EDX analysis, which clearly shows that aggregates visible on the surface are the composite nanoparticles, since it is in this area that the maximum Fe content of magnetite is observed (Fig. 7b,c).

## Conclusions

The present work describes a simple synthesis of magnetic nanoparticles with entrapped thrombin molecules, which showed a high hemostatic effect. To study the activity of THR@ferria NPs, coagulation time of blood plasma in glass and plastic cuvettes and hemostasis experiments on a model vessel were used, and cytotoxicity was evaluated for HELF and Hela cells, which showed no significant effect on cell survival. It is shown that with an identical amount of injected thrombin (free and entrapped) under conditions of normal fibrinogen concentrations in plasma, the time of blood coagulation is by a factor of 2 higher in the case of homogeneous enzymatic catalysis. As the fibrinogen concentration increases, the activity of the composite increases as well, while the activity of the native enzyme does not change significantly, yielding comparable values for coagulation times. The data obtained indicate that thrombin in the matrix retains the ability to hydrolyze four peptide bonds in the fibrinogen molecule, which results in the formation of a fibrin monomer, which is the basis of a blood clot. Using the model of an artificial blood vessel, it is also shown that the introduction of a magnetic colloid does not lead to systemic blood coagulation, but at the same time, due to the local focusing of nanoparticles in the bleeding zone upon application of an external magnetic field and additional injection of fibrinogen into the blood, it is possible to shorten the hemostasis time by a factor of 6.5. These results open up new prospects for the development of targeted delivery hemostatic agents for internal bleeding.

## Materials and Methods

### Materials

The hydrosol was prepared from iron (II) chloride tetrahydrate, iron (III) chloride hexahydrate and ammonia all obtained from Sigma-Aldrich. Thiazolyl blue tetrazolium bromide (MTT, 97.5%) was also obtained from Sigma-Aldrich. Dimethyl sulfoxide (DMSO) was obtained from VWR, and PBS Tablets were purchased from Gibco. Coomassie Brilliant Blue G-250 was obtained from Rosmedbio. Lyophilized human plasma and fibrinogen were obtained from Kvik. Thrombin (500 NIH units/mg) was obtained from Technology Standard. Donor blood was obtained from a 32-years old man. Blood obtained from a vein was poured into a tube with 5% sodium citrate solution (100 mL of blood, 10 mL of sodium citrate), then frozen at −18 °C and stored for 24 hours. Prior to the experiment, the blood was thawed at room temperature and heated to 37 °C in a water bath. The characteristics of the donor blood used to conduct the experiment with a model blood vessel are provided in the Supplementary Information. Experiments were approved by the institutional ethical committee (Mariinsky hospital, Russia) and were performed in accordance with the relevant guidelines and regulations. An informed consent was obtained for experiments involving human tissue samples.

### Synthesis of THR@ferria NPs

**Figure 8:**
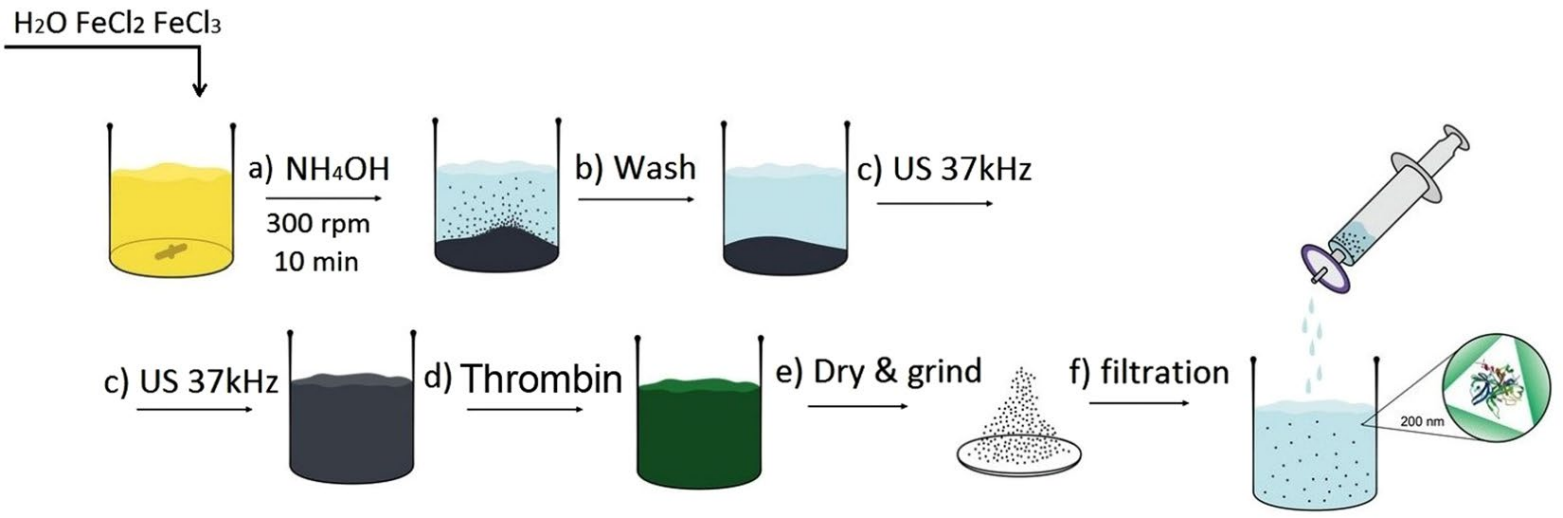
Scheme of synthesis for the hemostatic THR@ferria colloid. After mixing the Fe^2^^+^/Fe^3^^+^ salts, an ammonia solution is added (**a**) followed by washing the precipitate of the formed magnetite (**b**) and treating it ultrasonically to yield a stable hydrosol (**c**). Then, the solution of thrombin (**d**) is added to the sol and the system is condensed by removal of the solvent and physical gelation. The resulting composite with entrapped thrombin molecules is ground (**e**) and a hemostatic colloid is prepared by filtering the ground nanoparticles through a filter with a nozzle of 200 nm.

Scheme for synthesis of THR@ferria nanoparticles is shown in Fig. 8. Below we consider the various stages of synthesis in detail.

#### Synthesis of ferria NPs

Ferria hydrosol was prepared in a manner described in ref.^24^. 2.5 g FeCl_2_ 4 H_2_O and 5 g FeCl_3_.6 H_2_O (Fe^2+^/Fe^3+^ molar ratio of 0.7) were dissolved in 100 mL of deionized water while stirring (500 rpm) in anaerobic conditions. Then 12 mL of an aqueous ammonia solution was added to this solution at room temperature and constant stirring (Fig. 8a). A precipitate was collected using a magnet and washed with deionized water until neutral pH (Fig. 8b). The washed black precipitate was mixed with 100 mL of deionized water and ultrasonicated (37 kHz, 110 W) with constant stirring at 300 rpm (Fig. 8c). The ultrasonic treatment time was 120 min. The resulting magnetite sol was then cooled to room temperature. The mass fraction of the magnetite sol obtained was 2.2%.

#### Synthesis of THR@ferria composite

A Petri dish containing 165 *μ*L of the freshly prepared sol was treated with 0–12 *μ*L of a thrombin solution with 500 NIH units/mL (Fig. 8d) at room temperature. After mixing, the contents were dried in a vacuum desiccator for three hours. The resulting composite material was ground in an agate mortar (Fig. 1e), then suspended in deionized water and put through a Phenex 200 nm syringe filter to remove all particles with sizes larger than 200 nm (Fig. 8f). The resulting suspension was concentrated on a rotary evaporator under reduced pressure at 20 °C and suspended in saline solution with a final solid concentration of 1 wt. % of enzyme (1.3 NIH/mL solid) and used in experiments. To estimate the release of the enzyme, the same amount of ground composite was added to the quartz cuvette with 2 mL of Bradford solution and examined by a 595 nm absorption spectrum at 37 °C for 12 hours.

### MTT Assay

3-(4.5-dimethylthiazol-2-yl)-2.5-diphenyltetrazolium bromide (MTT) assay [6606682] was used to evaluate cytotoxic effects of synthesized NPs. Human embryonic lung fibroblasts (HELF) and HeLa cells obtained from Biolot (Saint-Petersburg, Russia) were maintained in Eagle’s medium (EMEM, Biolot), supplemented with 10% fetal bovine serum (FBS, Gibco) and gentamicin 50 *μ*g/mL (Biolot) at 37 °C and 5% CO2. The cells were subcultured regularly using trypsin/EDTA (Gibco). HELF and HeLa cells were seeded at densities of 104 or 5 × 103 cells/well respectively in 96-well plates. After 24 h cells were treated with different concentrations of as-made test compounds (12.5–200 *μ*g/mL) dissolved directly in culture media for 72 h. At the end of the treatment the medium with NPs was aspirated and 200 *μ*L of MTT solution in PBS (0.5 mg/mL) was added and incubated for 1.5 h at 37 °C in a CO2 incubator. Then the MTT solution was aspirated and the formazan crystals were solved by adding 200 *μ*L of DMSO with subsequent incubation for 15 minutes. The optical density was measured at 570 nm using a plate reading spectrophotometer Tecan Infinite 50^37^. Viability was calculated as the ratio of optical density obtained for each concentration of NPs and the average of that for the control wells (untreated cells).

### Coagulation tests

Synthesized THR@ferria NPs were tested for their ability to coagulate human plasma. Normalized plasma with a fibrinogen concentration of 2.8 mg/mL was used in the test. To carry out the experiment, 1.5 mL of human plasma was introduced into a polystyrene or glass cuvette with a total volume of 4 mL incubated at 37 °C, and 0.5 mL of a colloidal THR@ferria solution with an activity of 1.4 NIH/mL was added, followed by examination of the thrombus formation while measuring optical density of the solution at 500 nm. To change the effect of fibrinogen on the dynamics of thrombus formation, 500 *μ*L of fibrinogen with a concentration of 4 mg/mL was preliminarily injected into the plasma. 1.5 mL of human plasma was taken to carry out the control experiment, and after heating to 37 °C, 10 *μ*L of thrombin with an activity of 100 NIH units/mL was added to it.

### Hemostatic activity in model blood vessel

The hemostatic effect of the THR@ferria colloid was tested using a model vessel (installation scheme is shown in Fig. 8). The vessel is a transparent PVC tube with an internal diameter of 6 mm and a wall thickness of 1 mm connected to a peristaltic pump with a flow rate of 24 cm/s. Both ends of the vessel were put into a measuring glass containing 30 mL of citrated human blood. The glass of blood was placed on a water bath to maintain a temperature of 37 °C. The pump provided circulation of blood in the vessel with a volumetric flow rate of 68 cm^3^/s. Using a heated needle, the vessel was punctured to produce a hole with a diameter of 1 mm, then 1 mL of THR@ferria colloid was introduced into the glass with blood and concentrated with a neodymium magnet at the site of the vessel damage. The time and mass of blood loss was measured until bleeding had ceased.

To investigate the effect of the fibrinogen level on the coagulation dynamics, the blood was treated with a fibrinogen solution with a concentration of 20 mg/mL, bringing its total concentration to 3.9 mg/mL, after which the experiment was carried out as described above. For the control experiment, a sample of unmodified citrated blood circulating in the tube with a peristaltic pump at 37 °C was used, and during the experiment the time and volume of blood loss was similarly measured until bleeding had stopped completely.

#### Characterization methods

Surface area, pore volume and pore size distribution were investigated using Quantachrome Nova 1200e by nitrogen adsorption at 77 K and analyzed by the BET and BJH equations. Zeta potential was measured using a Photocor Compact-Z analyzer. The crystalline phase and crystallinity of the samples were measured by X-ray diffraction (Bruker D8 Advance) using Cu *Kα* radiation (*λ* = 1.54 Å). The samples were scanned for 2 h at a rate of 0.5 degrees per minute. SEM measurements were performed on a two-beam scanning microscope Tescan LYRA3 FEG. For measurements, the sample was deposited on the carbon tape, then carbon sputtering was carried out. The electronic image was obtained at low accelerating voltages (<20 kV) and low intensities of the probe, in order to diminish destruction of the samples. EDS measurements were performed by Oxford Instruments X-Max with a 150 mm^2^ sensor. The Raman spectra were recorded using the 633 nm He-Ne laser line on a Horiba Jobin-Yvon MicroRaman 300 spectrometer. The laser power on the samples employed was 0.030 mW and 0.344 mW, with 300 s and 120 s exposition per diffraction window, respectively. In all the measurements, 50x Olympus lens, hole of 500 *μ*m, slit of 100 *μ*m and a diffraction grid with 1800 grooves/mm were employed. The samples for transmission electron microscopy (TEM) were obtained by dispersing a small probe in ethanol to form a homogeneous suspension. Then, a suspension drop was coated on a copper mesh covered with carbon for a TEM analysis (FEI TECNAI G2 F20, at an operating voltage of 200 kV). Spectrophotometric measurements were carried out using an Agilent Cary HP 8454 Diode Array spectrophotometer with TEC. XPS spectra were recorded on an ESCALAB Xi X-ray Photoelectron Spectrometer.

### Electronic supplementary material

Supplementary Information
