# Author Correction: Thrombin@Fe_3_O_4_ nanoparticles for use as a hemostatic agent in internal bleeding

**DOI:** 10.1038/s41598-024-53918-z

**Published:** 2024-02-27

**Authors:** Emiliya M. Shabanova, Andrey S. Drozdov, Anna F. Fakhardo, Ivan P. Dudanov, Marina S. Kovaltschuk, Vladimir V. Vinogradov

**Affiliations:** 1 ITMO University, Laboratory of Solution Chemistry of Advanced Materials and Technologies, Lomonosov St. 9, 191002 St. Petersburg, Russian Federation; 2 Mariinsky Hospital, Regional Cardiovascular Center, Liteyny Ave. 56, 191054 St. Petersburg, Russian Federation

Correction to: *Scientific Reports* 10.1038/s41598-017-18665-4, published online 10 January 2018

This Article contains errors.

As a result of a typographical error the decrease of blood loss reported in the Results and Discussion was incorrectly reported,

“Even more outstanding data were obtained when measuring the mass of blood loss: 3.2 and 15.5 times smaller than those for the control sample with appropriate contents of fibrinogen in the blood.”

should read

“Even more outstanding data were obtained when measuring the mass of blood loss: 3.2 and 7.75 times smaller than those for the control sample with appropriate contents of fibrinogen in the blood.”

Additionally, Figure 6 reports averages of triplicates. This number of replicates is insufficient to calculate standard deviation; the error bars have been therefore removed and the corrected Figure 6 with an updated legend is shown below.

**Figure 6:**
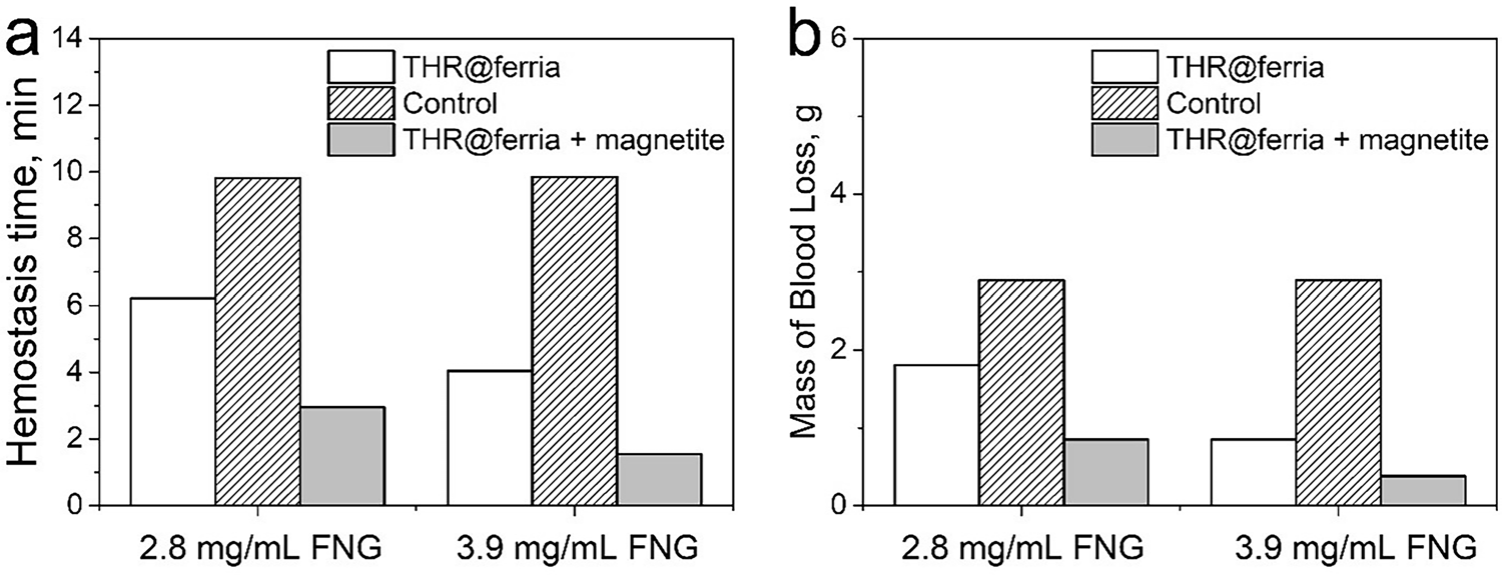
Time (**a**) and mass of blood (**b**) flowed out of the model blood vessel during an experiment on analyzing hemostatic activity. The measurements were carried out with different amounts of fibrinogen. The administration of hemostatic nanoparticles is shown to significantly reduce the time and mass of blood prior to hemostasis compared to the control, respectively when an external magnetic field is applied and the nanoparticles are concentrated accordingly. The results are an average of three measurements the values of which are given in the Tables 1S and 2S.

The numerical values underlying this figure are now shown in the updated Supplementary File (Tables 1S and 2S), which is appended to this notice values are presented in the updated Supplementary File.

### Supplementary Information

Supplementary Information.

