# Supplementary material for: Thrombin@Fe3O4 nanoparticles for use as a hemostatic agent in internal bleeding
Source: Sci Rep. 2018 Jan 10;8:233. doi: 10.1038/s41598-017-18665-4 (PMC5762673; doi:10.1038/s41598-017-18665-4)
Supplement: Supplementary file 1 — Supplementary Information [file 41598_2017_18665_MOESM1_ESM.doc]

Supplementary information for

**Thrombin@Fe_3_O_4_ nanoparticles for use as a hemostatic agent in internal bleeding**

**Emiliya M. Shabanova^1^, Andrey S. Drozdov^1*^, Anna F. Fakhardo^1^, Ivan P. Dudanov^1,2^, Marina S. Kovalchuk^2^ and Vladimir V. Vinogradov^1#^**

^1^ITMO University, Laboratory of Solution Chemistry of Advanced Materials and Technologies,

Lomonosov St. 9, 191002, St. Petersburg, Russian Federation

E-mail: [drozdov@scamt.ru](mailto:drozdov@scamt.ru), [vinogradov@scamt.ru](mailto:vinogradov@scamt.ru)

^2^Mariinsky Hospital, Regional Cardiovascular Center, Liteyny Ave. 56, 191054, St. Petersburg, Russian Federation


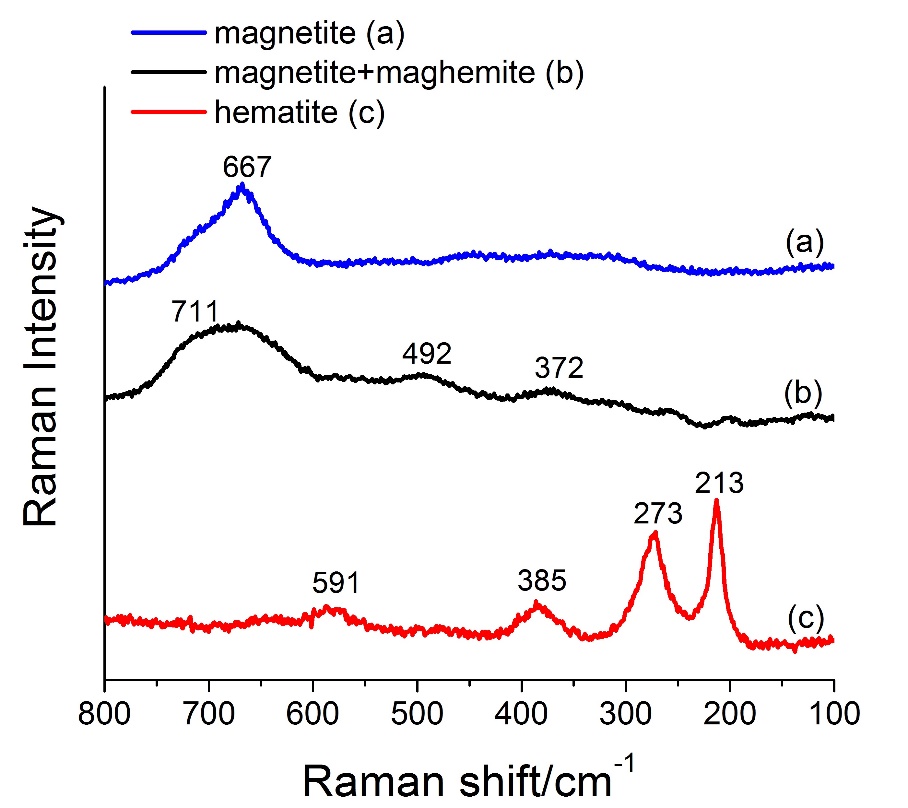


Figure 1S. Raman spectra of the used material. Line (a) (recorded at a 633 nm He-Ne laser power of 0.030 mW with acquisition time of 300 sec per diffraction window) shows the characteristic broad band of magnetite at 667 cm^-1^ [1S]. Further confirmation comes from the observations of the known phase transformations of magnetite [2S] under the Raman-laser measurement conditions: When the power was increased to 0.344 and 1.35 mW (acquisition times of 90 and 30 sec, respectively), then for the former, the characteristic maghemite bands at 711, 492 and 372 cm^-1^ are seen ( line b), and for the latter full oxidation to α-hematite is seen (line c) through the characteristic bands at 591, 385, 273, and 213 cm^-1^ [1S].


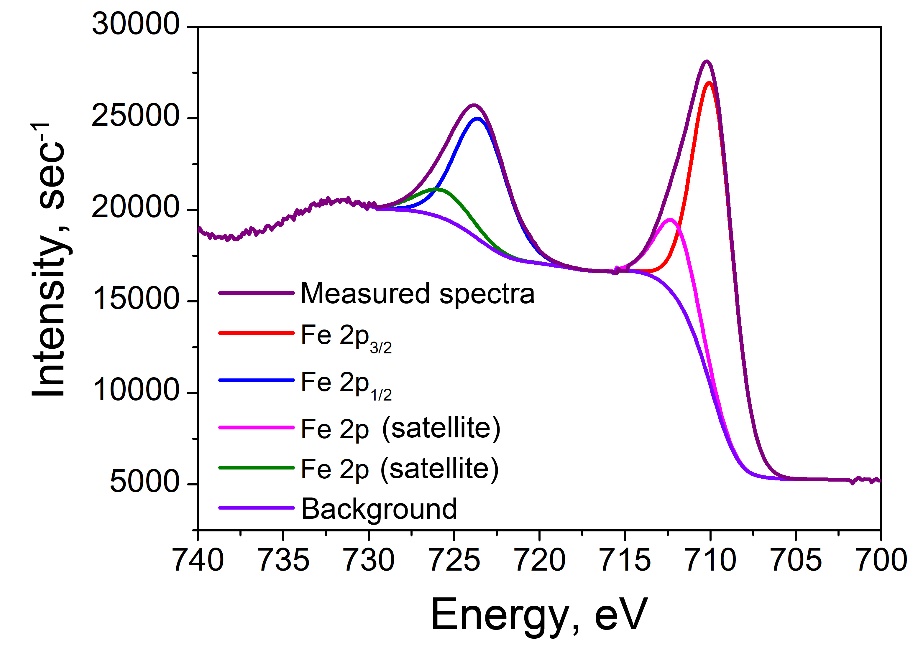


Figure 2S. XPS spectra of the used magnetite. XPS spectra is demonstrating peaks typical for magnetite at 723 and 711 eV. Absence of the satellite at 718 eV evidences the absence of maghemite phase in the material.


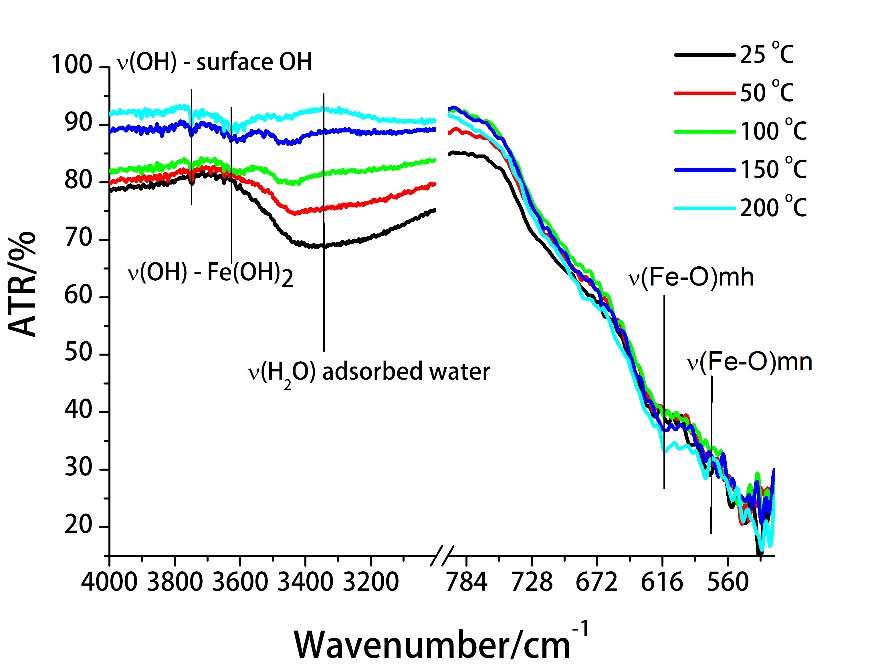


Figure 3S. ATR spectra of the magnetite sample. Analysis of the magnetite NPs surface shows peaks corresponding to valent vibration of OH surface groups. High content of OH groups on the NPs surface leads to high zeta-potential of the particles and results in the excellent colloidal stability.


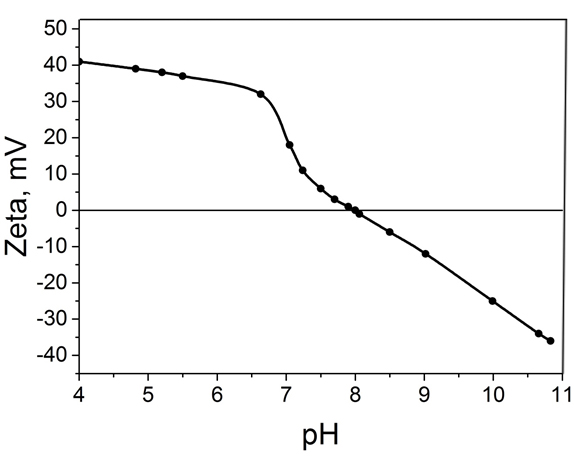


Figure 4S. Zeta potential of a magnetite matrix at different pH level. It is seen that the isoelectric point of the material corresponds to a pH of 8 and at neutral pH values the material is positively charged


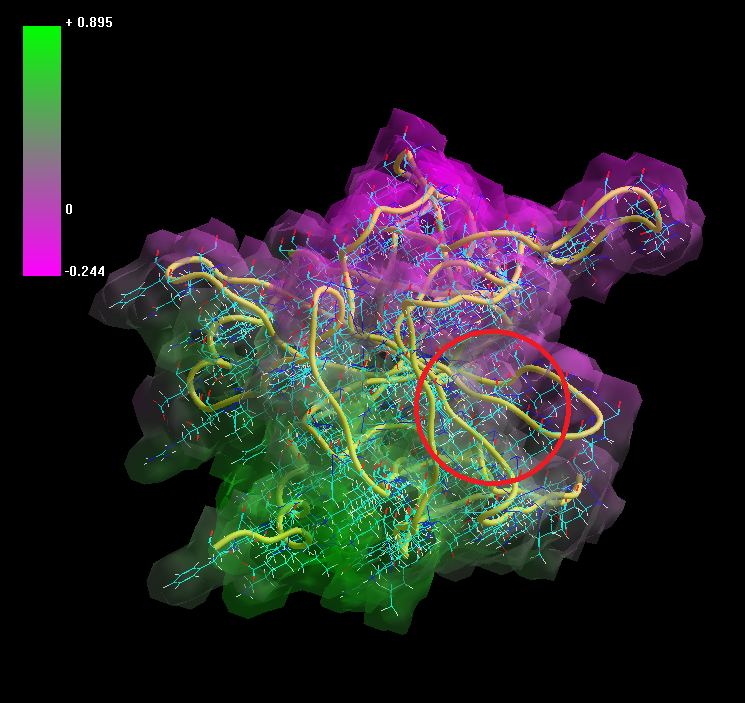


Figure 5S. Surface charge map of the thrombin molecule calculated in HyperChem 8.0.8 by MM+. Although thrombin molecule has net negative charge at pH 7.4 detailed examination shows positively-charged heparin-binding domain located near the catalytic center of the enzyme (marked with the red circle). When being entrapped into positively-charged magnetite matrix (IEP at pH 8) the thrombin is likely to orient in a manner to maximally distance this area from the matrix walls orienting its active center to the pores.
